# Effects of thermal water inhalation in chronic upper respiratory tract infections in elderly and young patients

**DOI:** 10.1186/s12979-016-0073-0

**Published:** 2016-05-05

**Authors:** Thea Magrone, Mauro Galantino, Nunzio Di Bitonto, Luisella Borraccino, Gerardo Chiaromonte, Emilio Jirillo

**Affiliations:** Department of Basic Medical Sciences, Neuroscience and Sensory Organs, University of Bari, Policlinico, Piazza Giulio Cesare 11, 70124 Bari, Italy; Thermal Water Station “Margherita di Savoia”, Margherita di Savoia, BAT Italy

**Keywords:** Ageing, Chronic upper respiratory tract infections, Cytokines, Thermal water inhalation

## Abstract

**Background:**

Chronic upper respiratory tract infections (cURTI) are very frequent illnesses which occur at any age of life. In elderly, cURTI are complicated by immunosenescence, with involvement of lung immune responsiveness.

**Results:**

In the present study, 51 elderly (age range: 66–86) and 51 young (age range 24–58) cURTI patients underwent a single cycle (two weeks) of inhalatory therapy with salt-bromide-iodine thermal water in the thermal station “Margherita di Savoia” (Margherita di Savoia, BAT, Italy). Peripheral blood serum cytokines and clinical assessment were performed before therapy (T0) and after six months (T1) and 12 months (T2) from inhalatory treatment. In both elderly and young patients, at baseline an increased release of T helper (h)1-related cytokines [interleukin (IL)-2 and interferon-γ] and of Th2-related cytokine (IL-4) was documented. Inhalatory treatment reduced the excessive secretion of all the above-cited cytokines. IL-10 values were above normality at all times considered in both groups of patients. In addition, an increase in IL-17 and IL-21 serum levels following therapy was observed in both groups of patients. Pro-inflammatory cytokine (IL-1β, IL-6, IL-8 and tumor necrosis factor-α) baseline values were lower than normal values at T0 in both elderly and young cURTI patients. Their levels increased following inhalatory treatment.

Clinically, at T2 a dramatic reduction of frequency of upper respiratory tract infections was recorded in both groups of patients.

**Conclusion:**

Thermal water inhalation is able to modulate systemic immune response in elderly and young cURTI patients, thus reducing excessive production of Th1 and Th2-related cytokines, on the one hand. On the other hand, increased levels of IL-21 (an inducer of Th17 cells) and of IL-17 may be interpreted as a protective mechanism, which likely leads to neutrophil recruitment in cURTI patients. Also restoration of pro-inflammatory cytokine release following inhalatory therapy may result in microbe eradication. Quite importantly, the maintenance of high levels of IL-10 during the follow-up would suggest a consistent regulatory role of this cytokine in attenuating the pro-inflammatory arm of the immune response.

## Background

Chronic upper respiratory tract infections (cURTI) are recurrent illnesses which involve nose, sinuses, pharynx, and larynx. Tonsillitis, pharyngitis, rhinitis, sinusitis, otitis, common cold and, sometimes, bronchial complications are the major clinical signs with periodical exacerbations in winter period [1]. Among viruses, the most common is Rhinovirus but also Adenovirus, Parainfluenza virus, Enterovirus and Respiratory Syncitial virus can be isolated from cURTI patients [2, 3]. Among bacteria, group A *Streptococcus* (*S.*), *S. pneumonia*, *S. pyogens*, *Haemophilus influenza* have frequently been detected in cURTI patients [2, 3]. Evidence has been provided that cURTI are very frequent diseases, involving millions of people annually and all ages are affected [4]. In elderly, cURTI are complicated by the decline of immune response in this period of life [5, 6]. In fact, multiple disorders of the innate and adaptive immunity have been described in ageing, thus increasing frequency of infections [7, 8].

Immunosenescence has recently been termed as senescent immune remodeling (SIR) [9, 10], and, in a recent review, Denkinger et al. [11–14] have pointed out the major immune alterations of T and B cells in elderly. With regard to modifications of innate immunity in elderly, a condition of low grade inflammation has been reported and termed as ‘inflammaging’ [15]. It has been demonstrated that ‘inflammageing’ also leads to hematopoietic stem cell (HSC) exhaustion and bone marrow failure [16], as also shown in mice following total body irradiation [17]. Conclusively, both low grade inflammation and HSC aging may be involved in SIR development [11].

With special reference to aged lungs, release of pro-inflammatory cytokines in the absence of a given stimulus may contribute to tissue destruction and loss of elasticity [18]. *Viceversa*, in the presence of injurious stimuli (*e.g.,* viruses and bacteria) elevated basal production of cytokines hampers release of pro-inflammatory mediators, as observed in aged and knockout animals [19–26]. Just recently, old dendritic cells (DCs) have been shown to contribute to chronic airway inflammation, releasing pro-inflammatory mediators, mostly tumor necrosis factor (TNF)-α, which affects primarily bronchial epithelial cell function in vitro, enhancing the epithelial barrier permeability, thus increasing susceptibility to infectious respiratory disease in ageing [27]. Finally, naïve CD4^+^ and CD8^+^ cells are reduced in aged animals and humans with an impaired conversion towards memory cells able to respond to new antigens in the context of respiratory tract [28, 29].

A series of papers have investigated the local immune response in cURTI, especially, with regard to chronic rhinosinusitis (CRS). Group 2 innate lymphoid cells (ILCs2) have been isolated in the nasal mucosa of CRS patients which correlated with T helper (h)-2 cell number, serum total IgE and allergic disease, such as asthma [30]. Furthermore, ILCs2 may account for polyp formation in these patients. In CRS with polyps, elevated levels of tissue interleukin (IL)-21 have been detected as products of Th1 and Th17 cells [31]. IL-21 correlated with disease severity, B cell production (IgG and IgA) and protracted inflammation of the mucosa. In patients with eosinophilic CRS, increased tissue levels of IL-25 have been detected [32]. Th2 and Th9 cells, which express IL-17RB mRNA levels and IL-25, seem to contribute to eosinophilia. Another study has reported production of IL-25, IL-33 and thymic stromal lymphoietin by sinus epithelial cells in response to microbial stimulation or airway injury in patients with CRS [33]. The above cited cytokine network seems to favor regional Th2-mediated inflammation. According to other researchers, the heavy infiltration of DCs in CRS with polyps may account for the Th2-mediated inflammation in this disease [34]. Finally, a defect of natural killer (NK) cells was detected in CRS patients refractory to treatment [35]. The inability of NK cells to degranulate and release interferon (IFN)-γ and TNF-α may account for the reduced cytotoxicity against target cells, even including infected cells.

Thermal water inhalation to patients with cURTI is a conventional treatment with long tradition [36]. The anti-inflammatory effects of thermal water are supported by current literature. Treatment with sulphurous thermal water in patients with rhinitis led to a reduction of polymorphonuclear cells in nasal mucosa [37]. Bromide-iodine thermal water decreased serum IgE and increased serum IgA in patients with allergic rhinitis [38]. Also in patients with chronic obstructive pulmonary disease (COPD) (a lower respiratory tract disease), inhalation of salt-bromide-iodine thermal water dramatically reduced neutrophil content in sputum [39]. These findings have suggested an anti-inflammatory effect exerted by thermal water inhalation in chronic respiratory disease but mechanisms of action have not yet been elucidated.

Aim of this study is to evaluate serum cytokines and clinical response of elderly and young cURTI patients to inhalation therapy with salt-bromide-iodine thermal water. Of note, cytokines for their pleiotropic effects seem to represent an appropriate biomarker of systemic inflammation in cURTI. Results will show that normalization of Th1 and Th2 exaggerated cytokine responses, increase in IL-21 production and recovery of impaired pro-inflammatory cytokine secretion likely lead to a dramatic clinical improvement in terms of partial or total abrogation of infectious recurrences. A hypothetical model of the mechanisms of action of thermal water inhalation is also illustrated.

## Results

Fifty-one elderly patients (mean age 68 years) and 51 young patients (mean age 48 years) were enrolled for this study.

### Th1-related cytokines

#### Elderly patients

**Fig. 1:**
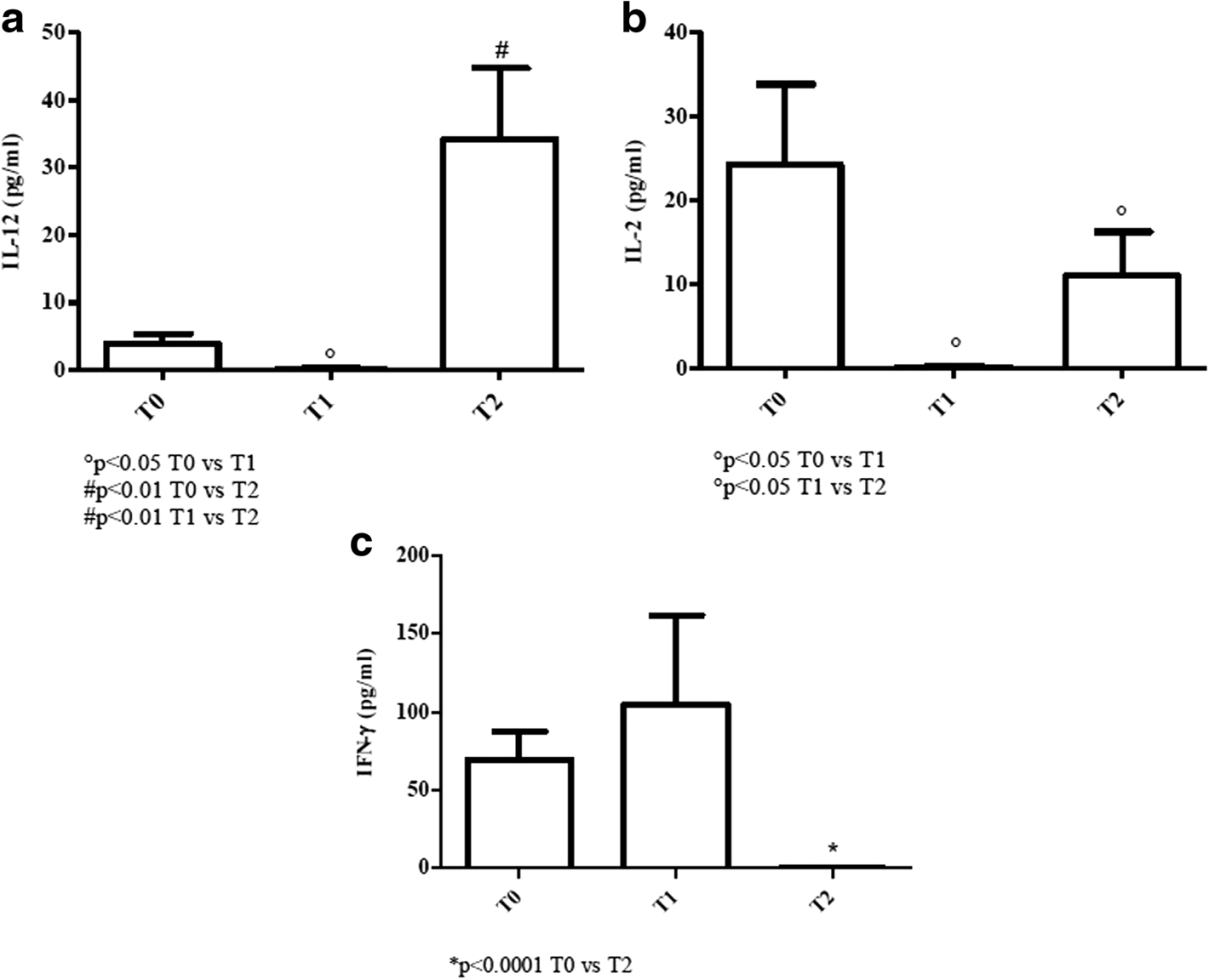
Determination of Th1-related cytokines in sera of cURTI elderly patients. Panel **a**: IL-12 serum level measurement; panel **b**: IL-2 serum level measurement; panel **c**: IFN-γ serum level measurement. Serum cytokines were assayed by flex set kit using a citofluorimetry (FACS Aria III) as described in material and methods. T0 = before treatment, T1 = after six months, T2 = after one year of treatment. Symbols indicate statistical analysis between samples performed by Student’s t-test (GraphPad Prism 5.0 software) and values with *p* < 0.05 were considered significantly

Serum levels of IL-12 significantly increased in elderly patients at T2 (*p* < 0.01), being values undetectable at T1 and above normality at T0 [values of young donors (controls): 0 pg/ml; values of elderly (controls): 0.8–3.8 pg/ml)] [Fig. 1a].

Serum levels of IL-2 were undetectable at T1 and significantly decreased at T2 (*p* < 0.05) in comparison to more elevated T0 values [values of young donors (controls): 0 pg/ml; values of elderly (controls): 4–20 pg/ml] [Fig. 1b].

A different profile was observed in the case of IFN-γ evaluation since its serum concentration dramatically dropped at T2 (*p* < 0.0001) when compared to higher amounts at T0 and T1 [values of young donors (controls): 0 pg/ml; values of elderly (controls): 0–7 pg/ml] [Fig. 1c].

#### Young patients

**Fig. 2:**
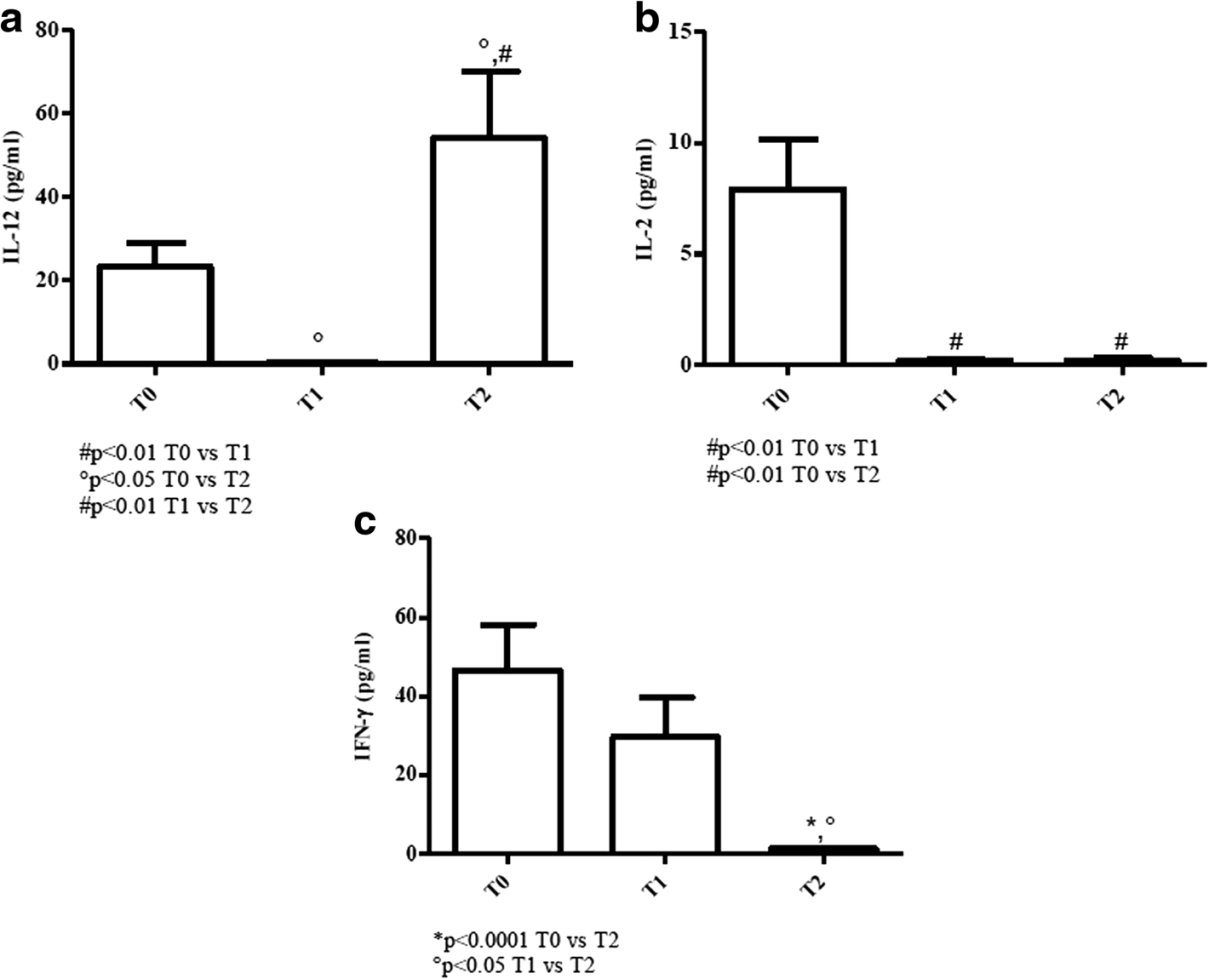
Determination of Th1-related cytokines in sera of cURTI young patients. Panel **a**: IL-12 serum level measurement; panel **b**: IL-2 serum level measurement; panel **c**: IFN-γ serum level measurement. Serum cytokines were assayed by flex set kit using a citofluorimetry (FACS Aria III) as described in material and methods. T0 = before treatment, T1 = after six months, T2 = after one year of treatment. Symbols indicate statistical analysis between samples performed by Student’s t-test (GraphPad Prism 5.0 software) and values with *p* < 0.05 were considered significantly

The response pattern of IL-12 was quite similar to that detected in the case of elderly patients with increasing cytokine values at T2 (*p* < 0.01) [Fig. 2a].

With regard to IL-2 serum levels, amounts of this cytokine significantly decreased at T1 and T2 (*p* < 0.01), being values almost undetectable [Fig. 2b].

IFN-γ serum level profile overlapped that noted in the case of elderly patients [Fig. 2c].

Conclusively, at T0 in both elderly and young cURTI patients Th1-related cytokines (IL-2 and IFN-γ) were higher than corresponding controls while values decreased at T2.

### Th2-related cytokine

#### Elderly patients

**Fig. 3:**
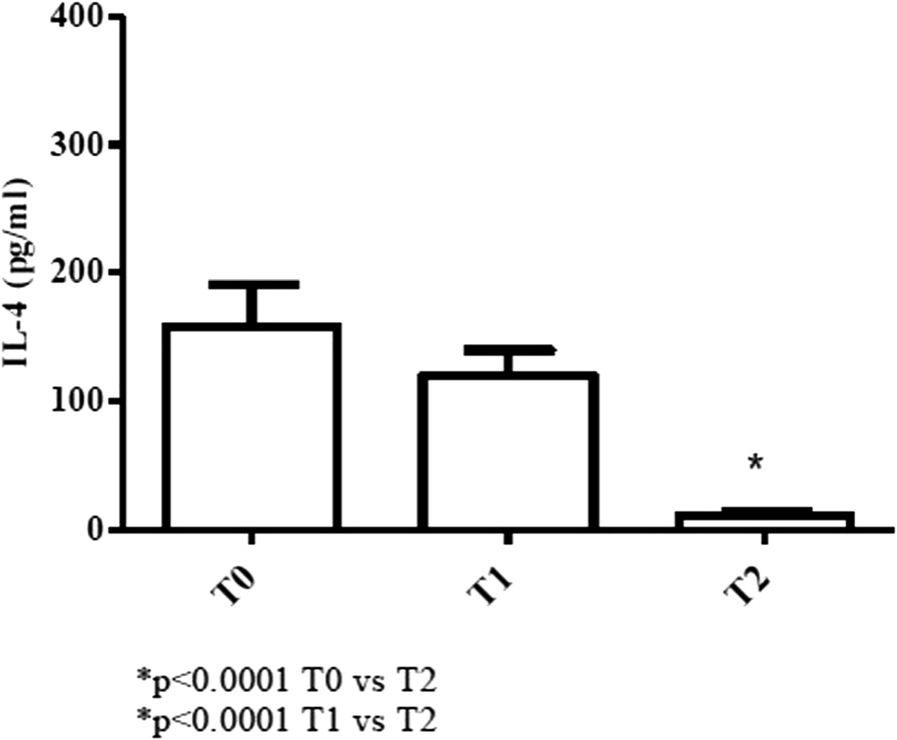
Determination of Th2-related cytokine in sera of cURTI elderly patients. IL-4 serum level measurement. Serum cytokines were assayed by flex set kit using a citofluorimetry (FACS Aria III) as described in material and methods. T0 = before treatment, T1 = after six months, T2 = after one year of treatment. Symbols indicate statistical analysis between samples performed by Student’s t-test (GraphPad Prism 5.0 software) and values with *p* < 0.05 were considered significantly

Elevated levels of IL-4, above normal values at T0 and T1, significantly decreased at T2 (*p* < 0.0001) [values of young donors (controls); 0.3–0.9 pg/ml; value of elderly (controls): 0–0.1 pg/ml] (Fig. 3).

#### Young patients

**Fig. 4:**
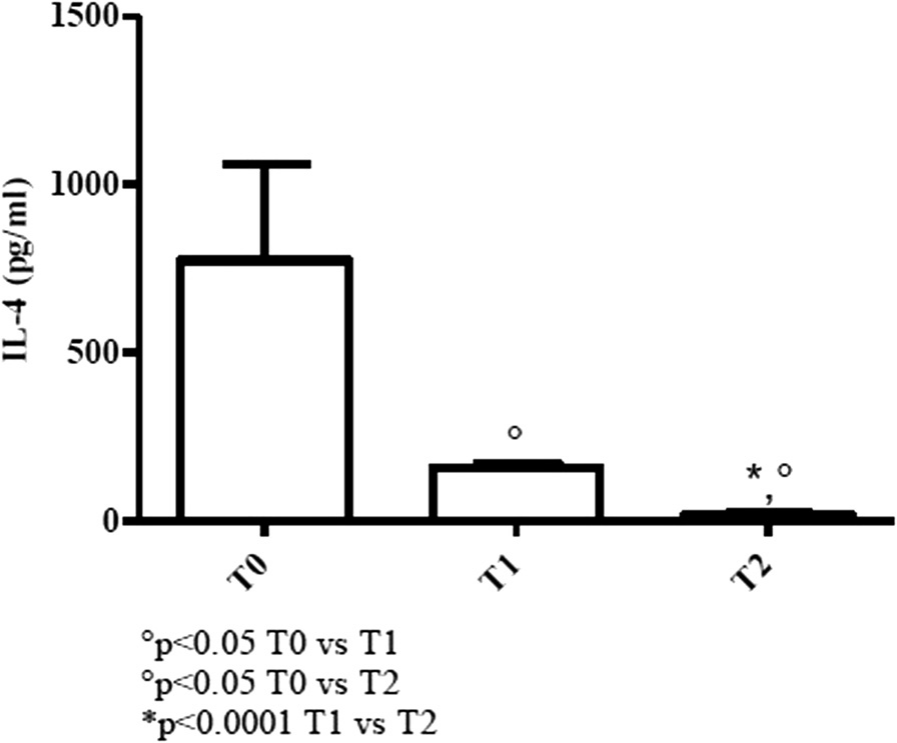
Determination of Th2-related cytokine in sera of cURTI young patients. IL-4 serum level measurement. Serum cytokines were assayed by flex set kit using a citofluorimetry (FACS Aria III) as described in material and methods. T0 = before treatment, T1 = after six months, T2 = after one year of treatment. Symbols indicate statistical analysis between samples performed by Student’s t-test (GraphPad Prism 5.0 software) and values with *p* < 0.05 were considered significantly

The above reported effects were more evident in young patients since IL-4 values, higher than the respective counterpart at T0, significantly dropped at T1 and T2 (almost undetectable) (*p* < 0.05) (Fig. 4).

In conclusion, reduction of IL-4, following inhalatory treatment, was more pronounced in young patients where baseline values were even higher than the elderly counterpart at T0.

### Evaluation of the IL-10/IL-17/IL-21 axis

#### Elderly patients

**Fig. 5:**
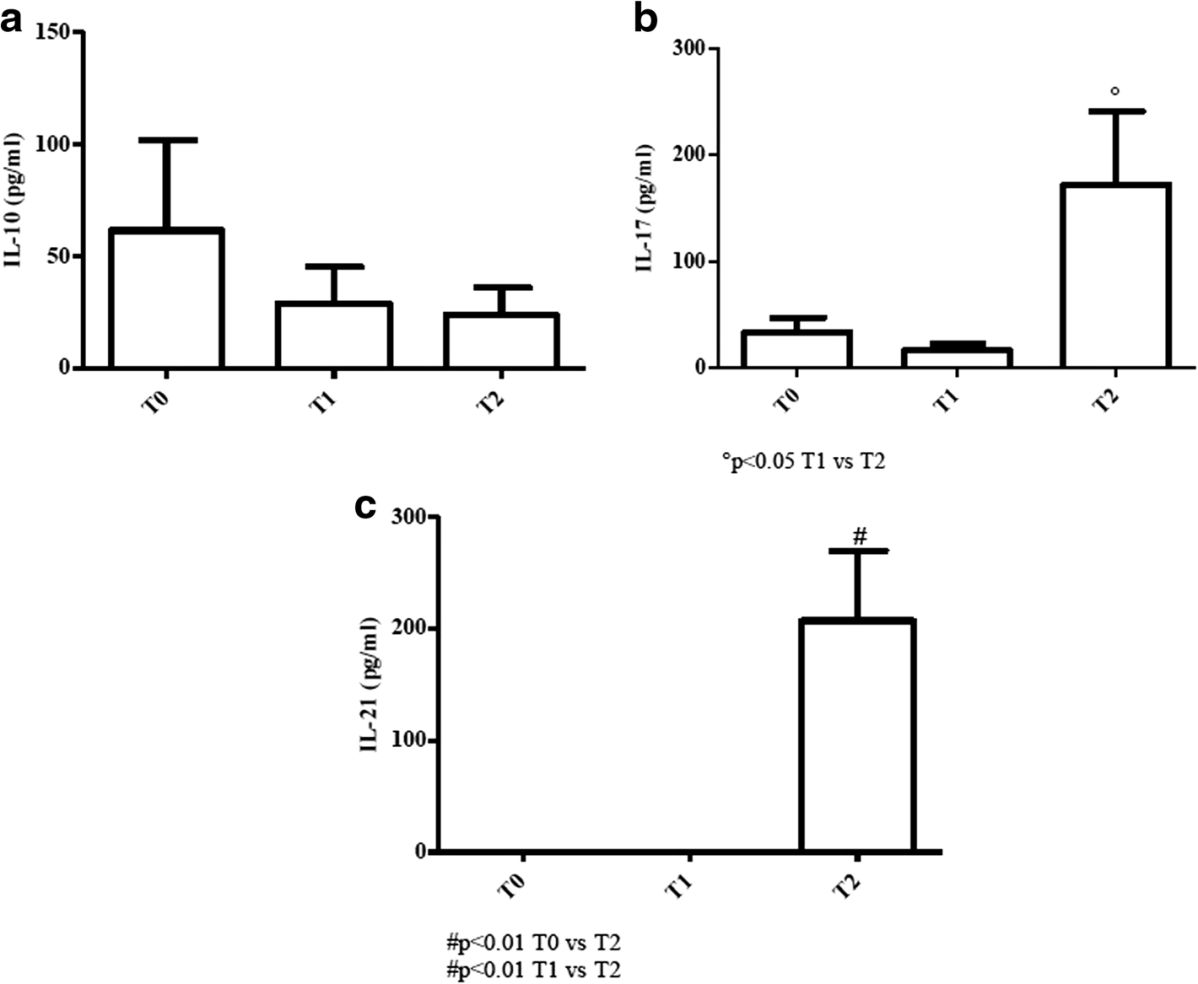
Determination of IL-10, IL-17 and IL-21 in sera of cURTI elderly patients. Panel **a**: IL-10 serum level measurement; panel **b**: IL-17 serum level measurement; panel **c**: IL-21 serum level measurement. Serum cytokines were assayed by flex set kit using a citofluorimetry (FACS Aria III) as described in material and methods. T0 = before treatment, T1 = after six months, T2 = after one year of treatment. Symbols indicate statistical analysis between samples performed by Student’s t-test (GraphPad Prism 5.0 software) and values with *p* < 0.05 were considered significantly

IL-10 values were above normality at all times considered even if a trend to decrease was observed at T1 and T2 [values of young donors (controls): 6–22 pg/ml; values of elderly (controls): 4–20 pg/ml] [Fig. 5a].

Conversely, values of IL-17 increased at T2 (*p* < 0.05) even above normal ranges [values of young donors (controls): 0 pg/ml; values of elderly (controls): 0 pg/ml] [Fig. 5b].

The previous finding was corroborated by a dramatic elevation of IL-21 (*p* < 0.01), a Th17 inducer (see discussion) at T2, while values at T0 and T1 were almost undetectable [values of young donors (controls): 6–18 pg/ml; values of elderly (controls): 0 pg/ml] [Fig. 5c].

#### Young patients

**Fig. 6:**
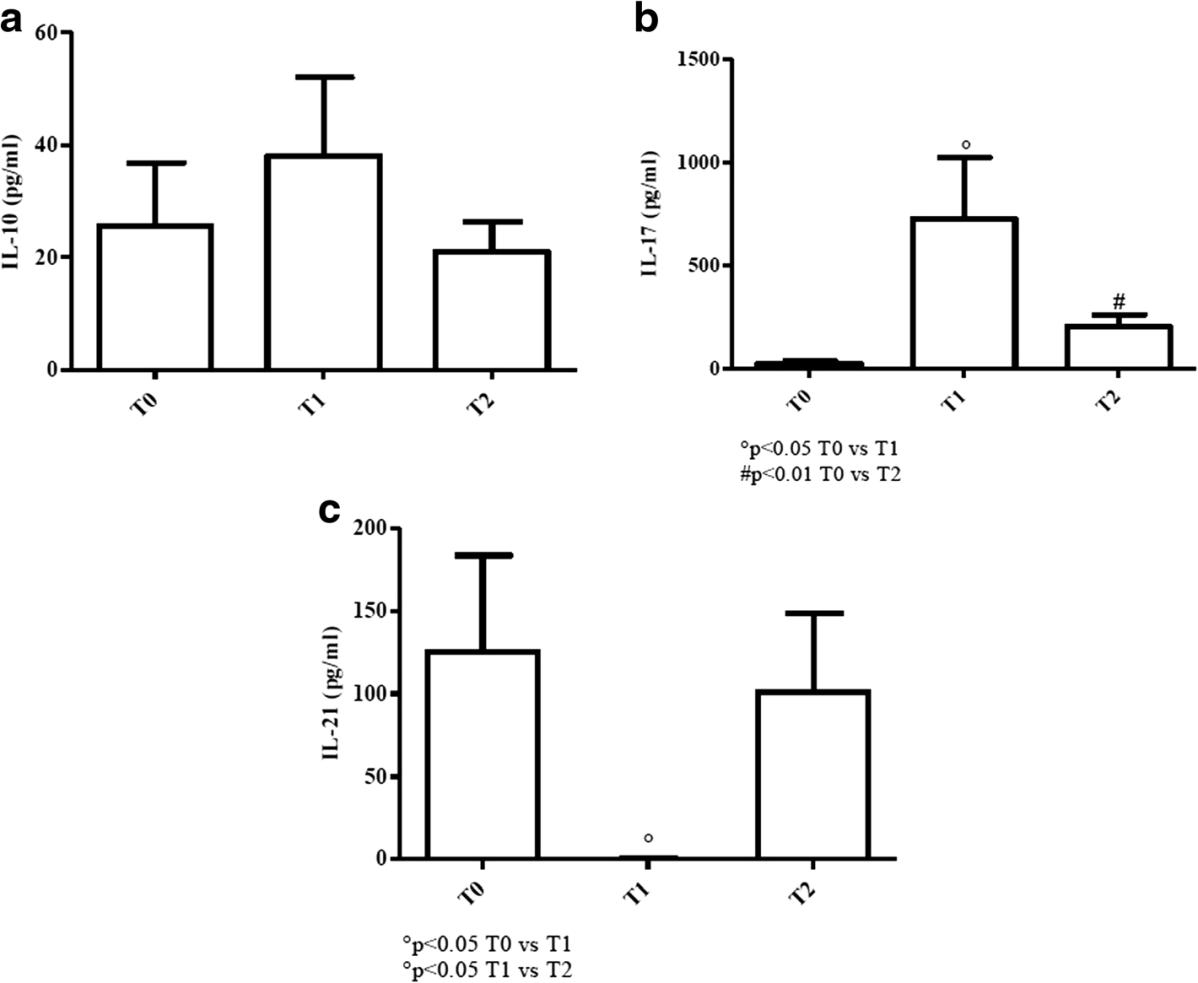
Determination of IL-10, IL-17 and IL-21 in sera of cURTI young patients. Panel **a**: IL-10 serum level measurement; panel **b**: IL-17 serum level measurement; panel **c**: IL-21 serum level measurement. Serum cytokines were assayed by flex set kit using a citofluorimetry (FACS Aria III) as described in material and methods. T0 = before treatment, T1 = after six months, T2 = after one year of treatment. Symbols indicate statistical analysis between samples performed by Student’s t-test (GraphPad Prism 5.0 software) and values with *p* < 0.05 were considered significantly

IL-10 profile was quite similar to that of elderly patients, being values above normality at all times considered [Fig. 6a].

IL-17 exhibited lower values at T0 which increased at T1 (*p* < 0.05) and T2 (*p* < 0.01) in a very significant manner. No differences were detected between T1 and T2 serum concentrations [Fig. 6b].

IL-21 amounts were higher at T0 than normal controls and elderly counterpart. Values were undetectable at T1 but significantly increased at T2 (*p* < 0.05) [Fig. 6c].

Taken together, these findings suggest that IL-10 response was very much elevated at all times in both groups of patients. In elderly, IL-21 increase at T2 seems to correlate with IL-17 elevation. However, the former cytokine was completely absent at T0 and T1. In young patients, IL-21 levels dropped at T1, but rose at T2.

### Evaluation of pro-inflammatory cytokines

#### Elderly patients

**Fig. 7:**
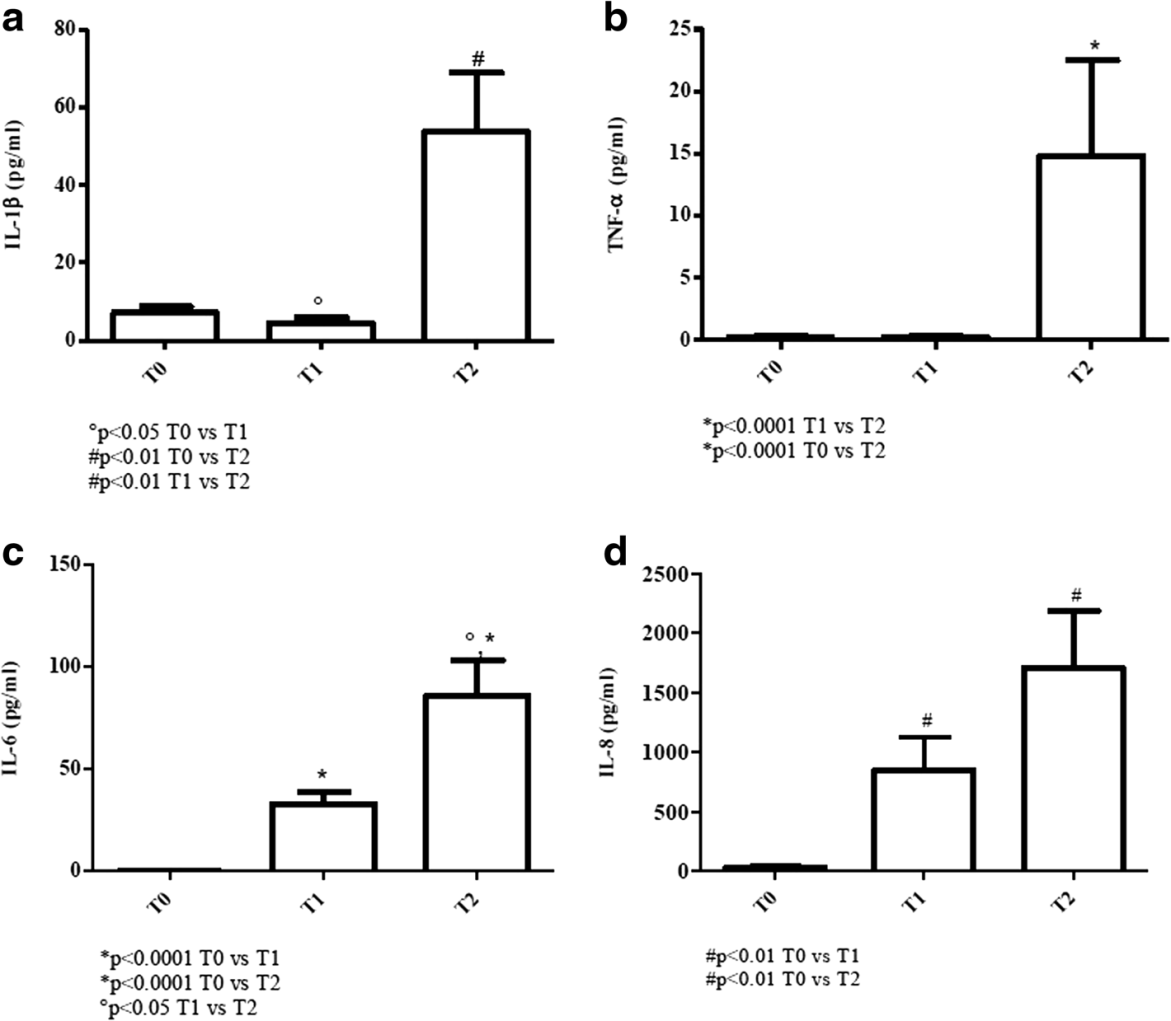
Determination of pro-inflammatory cytokines in sera of cURTI elderly patients. Panel **a**: IL-1β serum level measurement; panel **b**: TNF-α serum level measurement; panel **c**: IL-6 serum level measurement; panel **d**: IL-8 serum level measurement. Serum cytokines were assayed by flex set kit using a citofluorimetry (FACS Aria III) as described in material and methods. T0 = before treatment, T1 = after six months, T2 = after one year of treatment. Symbols indicate statistical analysis between samples performed by Student’s t-test (GraphPad Prism 5.0 software) and values with *p* < 0.05 were considered significantly

IL-1β values significantly increased at T2 (*p* < 0.01) in comparison to very low amounts at T0 and T1 [values of young donors (controls): 3–45 pg/ml; values of elderly (controls): 0,4–2,8 pg/ml] [Fig. 7a].

With regard to TNF-α, undetectable levels of this cytokine at T0 and T1 dramatically increased at T2 (*p* < 0.0001) [values of young donors (controls): 0,2–4,5 pg/ml; values of elderly (controls): 1–3,2 pg/ml] [Fig. 7b].

IL-6 was undetectable at T0 with a rise of values at T1 and T2 (*p* < 0.0001) [values of young donors (controls): 27–69 pg/ml; values of elderly (controls): 4–9 pg/ml] [Fig. 7c].

In the case of IL-8, values significantly increased at T1 and T2 (*p* < 0.01) when compared to undetectable levels at T0 [values of young donors (controls): 1382–7550 pg/ml; values of elderly (controls): 16–30 pg/ml] [Fig. 7d].

#### Young patients

**Fig. 8:**
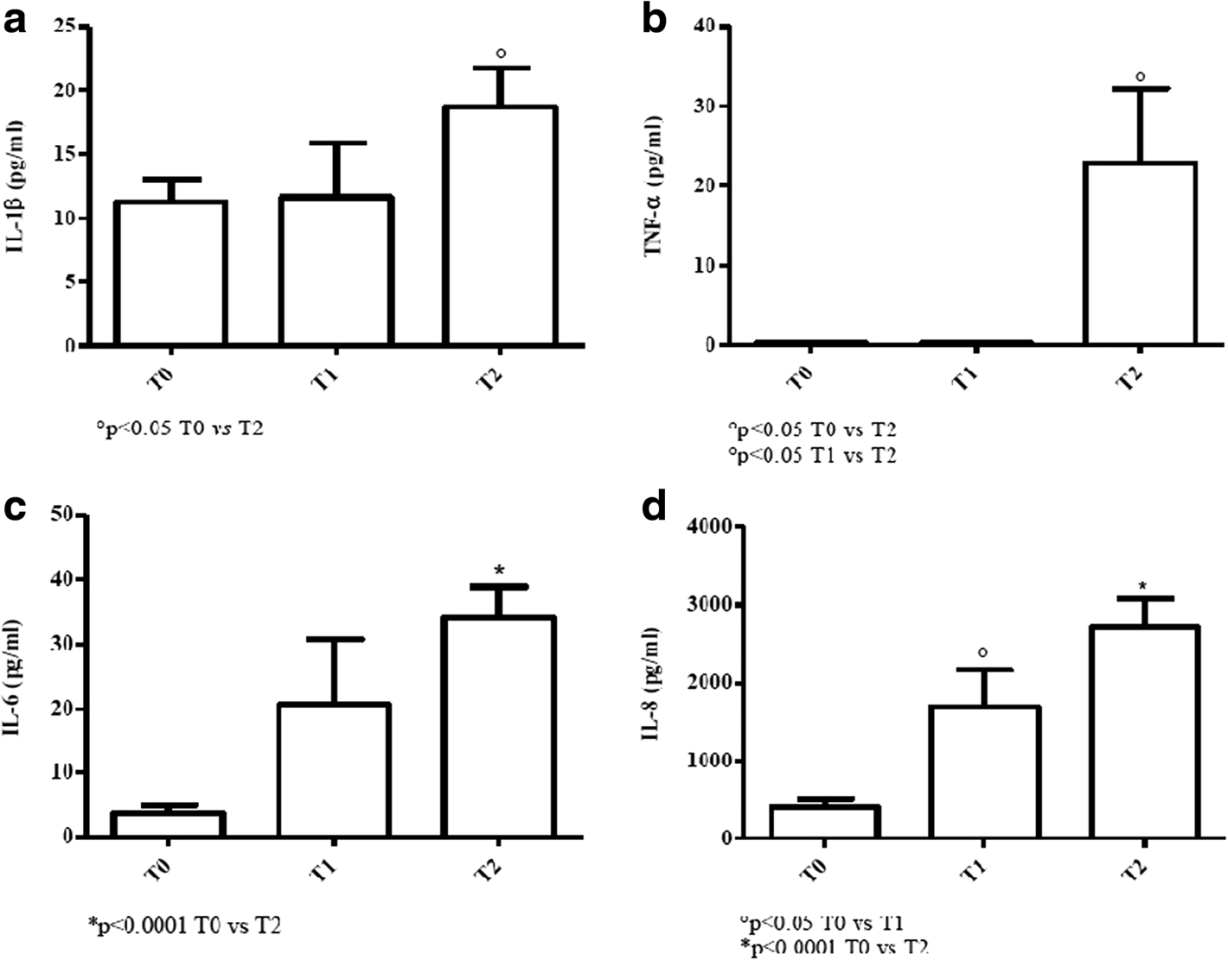
Determination of pro-inflammatory cytokines in sera of cURTI young patients. Panel **a**: IL-1β serum level measurement; panel **b**: TNF-α serum level measurement; panel **c**: IL-6 serum level measurement; panel **d**: IL-8 serum level measurement. Serum cytokines were assayed by flex set kit using a citofluorimetry (FACS Aria III) as described in material and methods. T0 = before treatment, T1 = after six months, T2 = after one year of treatment. Symbols indicate statistical analysis between samples performed by Student’s t-test (GraphPad Prism 5.0 software) and values with *p* < 0.05 were considered significantly

IL-1β baseline values were below normal ranges and an increase was noted at T2 (*p* < 0.05) [Fig. 8a].

TNF-α values were undetectable at T0 and at T1 but dramatically increased at T2 (*p* < 0.05) [Fig. 8b].

IL-6 progressively increased from T0 up to T2 (*p* < 0.0001) but values were below normal ranges [Fig. 8c].

IL-8 progressively increased at T1 and T2 in comparison to baseline values (*p* < 0.05 and *p* < 0.0001, respectively), with T2 values falling within normal ranges [Fig. 8d].

The overall findings suggest that in elderly and young cURTI patients the inflammatory cytokine pathway was impaired at T0 but restored by inhalatory treatment at T1 and T2 except for IL-6.

### Clinical evaluation

Among 48 elderly cURTI patients, 33 attained a full recovery of clinical manifestations at T2 (no infectious episodes) in comparison to 3 events at T0. In 15 of them, only one episode was recorded at T2 when compared to 2.2 infectious events at T0.

In the young patient counterpart, 29 individuals exhibited a full recovery (no episodes at T2 *vs* 4 events at T0). In the remaining 19 cases, 1.6 episodes at T2 *vs* 4 events at T0 were recorded.

Results are summarized in Table 1.

**Table 1:** Evaluation of frequency of infectious episodes at T0 and T2 in elderly and young patients with cURTI

	Number patients	Mean of episodes at T0	Mean of episodes at T2
Elderly subjects			
Complete response	33	3	0
Partial response	15	2.2	1
Young subjects			
Complete response	29	4	0
Partial response	19	4	1.6

## Discussion

cURTI are very common pathologies which afflict both young and old individuals. In elderly, the decline of immune response may aggravate the clinical course of cURTI because of the prolonged persistence of viruses and bacteria in the lung [40]. For the above reasons, in the present study we have compared the immune and clinical responses of elderly cURTI patients to those of a younger counterpart following inhalatory therapy with bromide-iodine thermal water.

Serum cytokine assessment immediately before and after six months and one year after a single cycle of inhalatory therapy has allowed monitoring both innate and adaptive arms of the immune response in both groups of patients.

It is well known that in response to a microbial stimulus, DCs acting as antigen presenting cells releas**e** IL-12 for the polarization of Th1 cells [41–43]. On these bases, we have evaluated IL-12 in sera from both groups of cURTI patients. IL-12 values are above normal ranges in both elderly and young patients with a significant rise at T2. In elderly, Th1-related cytokines are released in excess as in the case of IL-2 even if, a trend to its decrease can be observed at T2. In the case of IFN-γ, elevated values at T0 and T1 significantly decrease at T2. This pattern of response is similar to that seen in young patients, thus suggesting that in cURTI no differences are present between older and young patients in relation to Th1 function.

Quite interestingly, IL-4 evaluation in elderly and young patients, as a product of Th2 cells [44, 45], indicates that its release is above normality at T0 and at T1 but decreases at T2.

All together, our data suggest that both cellular and humoral adaptive immunity is involved in cURTI and no major differences can be seen between older and young patients in terms of functional changes following therapy.

To better clarify the immunological effects exerted by the inhalatory treatment, the role of IL-10 needs to be elucidated. IL-10, an anti-inflammatory cytokine, is predominantly produced by regulatory T cells as well as by Th2 cells [46, 47]. Both in elderly and young cURTI patients, values of IL-10 are higher than controls and, it is likely to hypothesize that inhalatory treatment contributes to maintain its continuous production. In fact, reduction of major cytokines such as IL-2, IL-4 and IFN-γ may result from the IL-10-mediated down-regulation during treatment. This effect may also lead to a reduction of lung and systemic immunopathology, attenuating the prolonged inflammatory effects of IFN-γ [48] as well as the IL-4-mediated allergic-inflammatory respiratory responses [49]. Mostly, in young cURTI patients, reduced levels of IL-2 thanks to inhalatory treatment may contribute to a diminished expansion of Th1 and Th2 antigen-specific cells [45].

In this framework, we have evaluated IL-21 release since it seems to act as an inducer of Th17 cells which, in turn, are able to produce another wave of IL-21 [50, 51]. The increased levels of IL-21 and IL-17 at T2 in both groups of patients may be interpreted as a protective mechanism rather than detrimental. In fact, evidence has been reported that in animal models IL-17 plays a critical role in host defence against bacteria in the airways [52]. Protection seems to depend on Th17-mediated release of neutrophil-mobilizing cytokines and attraction of neutrophils to antigenic sites, where they neutralize microorganisms. In this connection, evidence has been provided that reduced release of IL-17 may increase susceptibility to bacterial infections in smokers with COPD [52].

Evaluation of pro-inflammatory cytokines in cURTI patients has led to results of pathogenic as well as clinical relevance. In fact, especially in elderly patients all serum cytokine amounts (IL-1β, IL-8, TNF-α and IL-6) are reduced but they increase following inhalation therapy, even including IL-6, which, however, still remains below normality. In the younger patient counterpart, values of IL-1β, IL-6, IL-8 and TNF-α are below normality, with an increase of levels at the end of therapy. Inflammatory responses mediated by cytokines are of paramount importance for microbe eradication [53] and, in both cURTI patients restoration of the pro-inflammatory pathway by inhalation therapy may contribute to clinical improvement (see below). Several cell types in the lung or systemically can produce the above mediators, even including, monocytes/macrophages, DCs, neutrophils and epithelial cells. It is hard to identify in this work the actual cellular source of pro-inflammatory cytokines, but we are tempted to hypothesize that neutrophils activated by IL-17 can act as important players in the restoration of the pro-inflammatory pathway in cURTI. On the other hand, IL-10 elevated levels may contribute to the containment of excessive pro-inflammatory functions by neutrophils, assuming that these cells are the actual producers of pro-inflammatory cytokines.

Clinically, inhalatory treatment seems to be very effective in cURTI patients, as indicated by absence or reduction of infectious episodes at T2. Of note, in the young patients response to treatment seems to be more effective. The clinical improvement is long-lasting continuing after one year from the initial treatment. In this connection, homeostasis of the immune response and recovery of impaired pro-inflammatory cytokine release, once initiated by inhalation treatment, seems to persist with the time and contributes to anti-microbial protection. By the way, we are dealing with chronic patients also exposed to environmental antigens (pollutants, smoke, various allergens) and, therefore, it is not surprising to uncover in this study a condition of systemic involvement of the peripheral blood immune response.

Actually, cURTI are sustained by a complex of microbes, involving both respiratory viruses and co-infecting bacteria, thus leading to involvement of other organs, even including gut (the so-called lung-gut axis) [54]. Chronicity of mucosal inflammation also leads to alteration of gut microbiota with passage of bacterial products to circulation, thus favoring a detectable systemic inflammation [55].

In this direction, further studies should consider a longer follow-up of patients in terms of immune system monitoring and clinical evaluation, also including the assessment of gut microbiota [56, 57].

## Conclusion

**Fig. 9:**
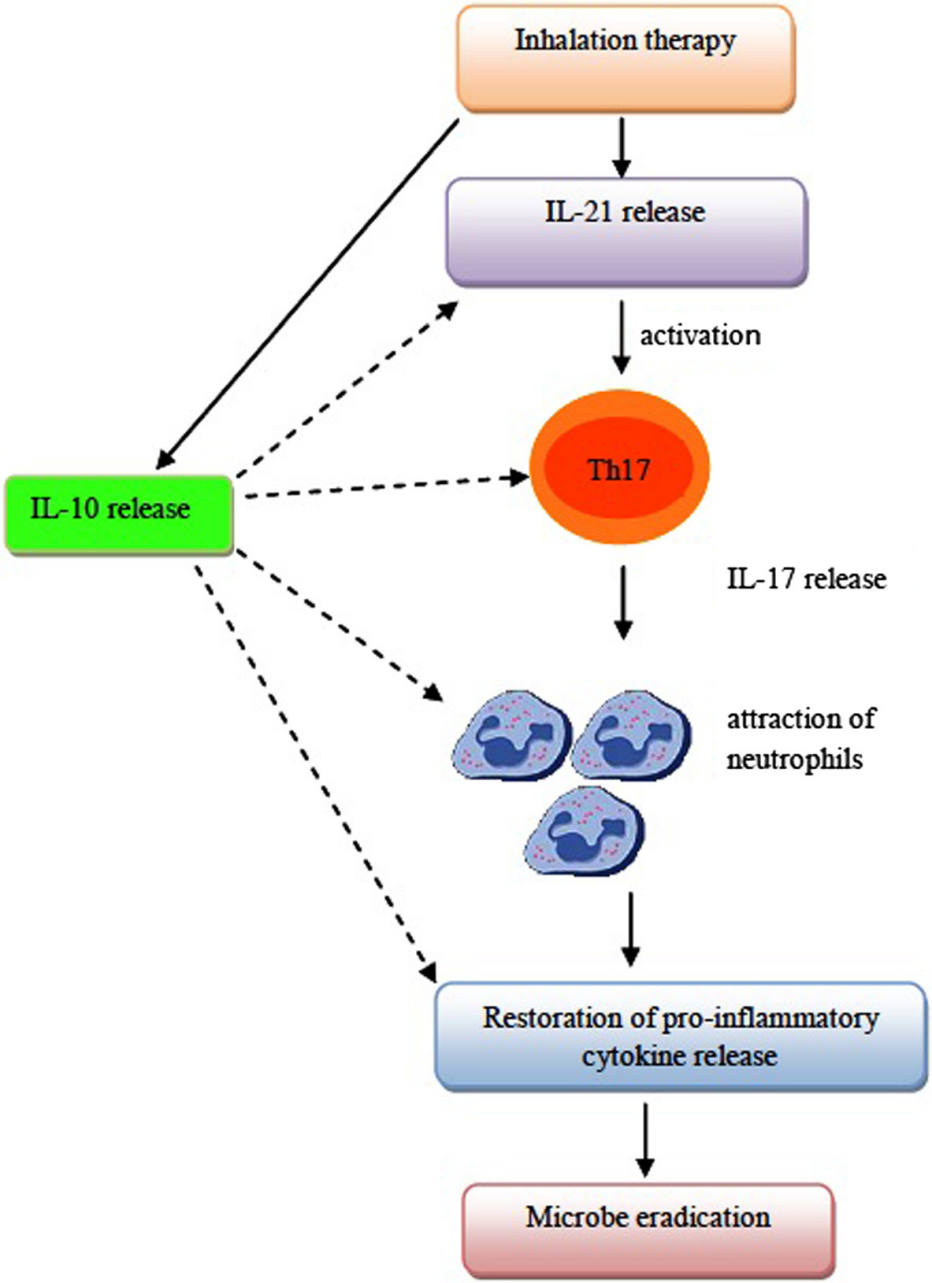
Hypothetical model of immune modulation exerted by thermal water inhalation in elderly and young cURTI patients. The key result is represented by the increased levels of IL-21 which may lead to an augmented secretion of IL-17. Subsequent recruitment of neutrophils may also restore reduced serum levels of pro-inflammatory cytokines in patients. Consistent levels of IL-10, mainly produced by T regulatory cells, may restrain the potential induction of an exaggerated inflammatory pathway

The reduced frequency of infectious episodes in both groups of cURTI patients following thermal water inhalatory treatment may rely on the modulation of immune responses in these patients. Our hypothesis is that increased levels of IL-21 may trigger Th17 mediated release of IL-17. Subsequent attraction of neutrophils may lead to an inflammatory wave of cytokines which restore the otherwise impaired innate immunity in cURTI patients. The consistent release of IL-10 may restrain the exaggerated inflammatory response at respiratory and systemic levels (see Fig. 9).

## Methods

### Patient enrollment

In patients with cURTI, before inhalatory treatment, specifc diagnosis was made in public or private hospitals. Major clinical manifestations of the upper respiratory tract were represented by tonsillitis, pharyngitis, rhinitis, sinusitis, otitis, common cold and, sometimes, bronchitis. Patients with a diagnosis of cURTI (51 aged persons and 51 young individuals) were randomly assigned to undergo inhalation treatment for 2 weeks in the thermal water station “Margherita di Savoia” (Margherita di Savoia, BAT, Italy). In both groups of patients, 3 dropouts were recorded.

The main water solutes are the following: Br^−^ (0.045 g/l); Cl^−^ (156 g/l) Na (85 g/l), SO4^−^ (36 g/l); I^−^(0.0005 g/l); Ca (0.38 g/l). Thermal water was free from bacterial contamination, and had a pH equal to 7.85.

Table 2 illustrates patient demographic information and relevant clinical data. Patients with allergic disease or dysmetabolic syndrome or had suffered a respiratory infection or taken immunosuppressive drugs in the last month were excluded. All participants gave an informed consent and the study design was approved by the Thermal Station ethical committee.

**Table 2:** Salient demographic and clinical features in elderly and young patients with cURTI

	Elderly		Younger	
Mean Age	68		48	
Sex	27 F	24 M	28 F	23 M
Frequency of infectious episodes/year	3 (T0)		4(T0)	

### Inhalatory treatment

Nebulised thermal water under form of microparticles (2–10 μm) prewarmed at 37 °C was administered through the rhino-pharynx tubal unit once a day for 10 min for 2 weeks at 2–3° Baumè density.

### Laboratory and clinical follow up

Peripheral blood from all patients was taken at enrollment (T0), T1 (after 6 months) and T2 (after one year). Sera were kept at −30 °C before use. As internal controls, 30 sera were obtained from young healthy donors (age range: 33–52) enrolled at the Blood Bank from Bari Polyclinic Hospital (Bari, Italy). Other 20 sera were obtained from healthy free-living elderly individuals (age range: 68–81) following an informed consent.

**Fig. 10:**
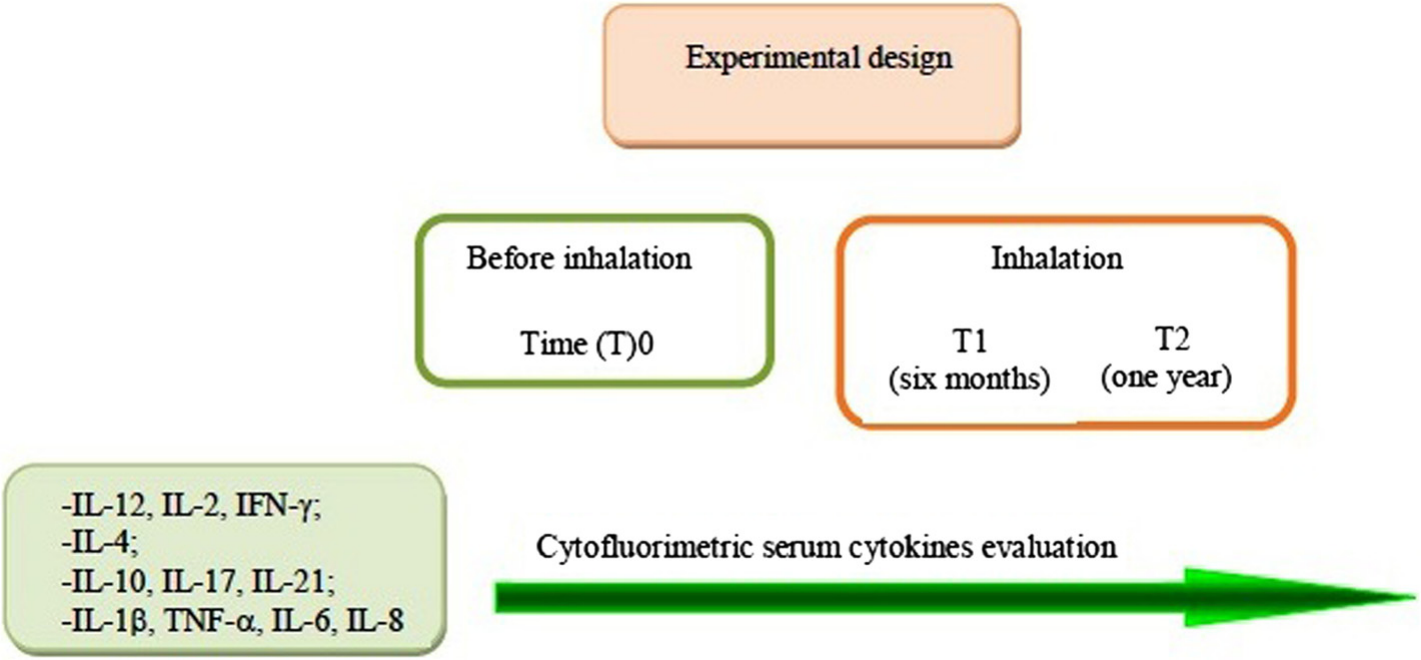
Experimental design related to the inhalation therapy and serum cytokine assay in elderly and young cURTI patients. Inhalation therapy with thermal water was based on a single cycle for two consecutive weeks. Serum cytokine evaluation was performed before treatment (T0) and after six months (T1) and one year (T2) from inhalatory therapy

Experimental design is depicted in Fig. 10.

Numbers of infectious episodes were recorded through a specific questionnaire which each patient received at the admission to the study.

### Cytofluorimetric evaluation of serum cytokines

Serum cytokine determination was performed by a FACSAria III cytofluorimetry (Becton & Dickinson, Milan, Italy) utilizing the CBA Flexset kit (Becton & Dickinson). The FCAP software was used and cytokines were expressed in pg/ml.

### Statistical analysis

Statistical analyses were performed by Student’s t-test (GraphPad Prism 5.0 software) and values with *p* < 0.05 were considered significantly.

## Abbreviations

COPD: chronic obstructive pulmonary disease
CRS: chronic rhinosinusitis
cURTI: chronic upper respiratory tract infections
DCs: dendritic cells
HSC: hematopoietic stem cell
IFN: interferon
IL: interleukin
ILCs2: innate lymphoid cells 2
SIR: senescence immune remodeling
TNF: tumor necrosis factor
